# Protein timing and its effects on muscular hypertrophy and strength in individuals engaged in weight-training

**DOI:** 10.1186/1550-2783-9-54

**Published:** 2012-12-14

**Authors:** Matthew Stark, Judith Lukaszuk, Aimee Prawitz, Amanda Salacinski

**Affiliations:** 1 School of Family, Consumer, and Nutrition Sciences. Northern Illinois University, DeKalb, IL, USA; 2 The Department of Kinesiology and Physical Education, Northern Illinois University, DeKalb, IL, USA

**Keywords:** Protein timing, Muscular hypertrophy, Muscular strength, Body composition, Whey protein, Milk protein, Protein synthesis

## Abstract

The purpose of this review was to determine whether past research provides conclusive evidence about the effects of type and timing of ingestion of specific sources of protein by those engaged in resistance weight training. Two essential, nutrition-related, tenets need to be followed by weightlifters to maximize muscle hypertrophy: the consumption of 1.2-2.0 g protein.kg ^-1^ of body weight, and ≥44-50 kcal^.^kg^-1^ of body weight. Researchers have tested the effects of timing of protein supplement ingestion on various physical changes in weightlifters. In general, protein supplementation pre- and post-workout increases physical performance, training session recovery, lean body mass, muscle hypertrophy, and strength. Specific gains, differ however based on protein type and amounts. Studies on timing of consumption of milk have indicated that fat-free milk post-workout was effective in promoting increases in lean body mass, strength, muscle hypertrophy and decreases in body fat. The leucine content of a protein source has an impact on protein synthesis, and affects muscle hypertrophy. Consumption of 3–4 g of leucine is needed to promote maximum protein synthesis. An ideal supplement following resistance exercise should contain whey protein that provides at least 3 g of leucine per serving. A combination of a fast-acting carbohydrate source such as maltodextrin or glucose should be consumed with the protein source, as leucine cannot modulate protein synthesis as effectively without the presence of insulin. Such a supplement post-workout would be most effective in increasing muscle protein synthesis, resulting in greater muscle hypertrophy and strength. In contrast, the consumption of essential amino acids and dextrose appears to be most effective at evoking protein synthesis prior to rather than following resistance exercise. To further enhance muscle hypertrophy and strength, a resistance weight- training program of at least 10–12 weeks with compound movements for both upper and lower body exercises should be followed.

## Review

### Purpose

Individuals who engage in resistance weight training, whether as competitive weightlifters or to promote optimal physical outcomes, would benefit by knowing the ideal nutritional intake protocol needed to maximize muscle hypertrophy and strength. The type, timing (pre/post workout) or amount of protein intake required to meet strength-training goals may not be clear to weightlifters or their trainers. The purpose of this review was to determine whether past research provides conclusive evidence about the effects of type and timing of ingesting specific protein sources by those engaged in resistance weight training. The review targets the effects of intake and timing of the following protein sources on physical outcomes: whey, casein, milk, soy and essential amino acids.

### Protein and calorie intake

For maximal muscle hypertrophy to occur, weightlifters need to consume 1.2-2.0 grams (g). protein kilogram. (kg)^-1^ and > 44–50 kilocalories (kcal)^.^kg^-1^ body weight daily [1-9]. This is considerably higher than the recommended dietary allowance (RDA) for protein (currently 0.8 g^.^kg^-1^) which meets the needs of 97.5% of all healthy adult Americans not engaged in weightlifting with the intent of gaining muscle mass [8]. Table 1 summarizes ranges for protein intake for weightlifters based on previous literature reviews.

**Table 1:** Summary of protein requirements for weightlifters

**Research study**	**Recommendation for protein intake**	**Type of study**
Lemon [1]	1.6-1.7 g^.^kg^-1^	Review of literature
Lemon et al. [2]	12-15% total energy intake	Review of literature
Kreider [3]	1.3-1.8 g^.^kg^-1^	Review of literature
Phillips [4]	12-15% total energy intake	Review of literature
Lemon [5]	1.6-1.8 g^.^kg^-1^	Review of literature
Lemon [6]	1.5-2.0 g^.^kg^-1^	Review of literature
Campbell et al. [7]	1.4-2.0 g^.^kg^-1^	Review of literature

### Leucine and muscle protein synthesis

The leucine content of a protein source has an impact on protein synthesis, and affects muscle hypertrophy [10-15]. This section details the role of leucine in protein synthesis to illustrate its importance in the process.

Protein synthesis occurs when methionyl-transfer ribonucleic acid (methionyl-tRNA) binds to a eukaryotic small ribosomal subunit (40S ribosomal unit) resulting in the formation of a pre-initiation complex (43S pre-initiation complex) [16]. This initial step is mediated by eukaryotic initiation factor 2 (eIF2) [16]. The 43S complex subsequently binds to messenger ribonucleic acid (mRNA) near the cap structure. After successful engagement of the 43S pre-initiation complex to RNA, the molecule eukaryotic initiation factor 5 (eIF5) removes eIF2 while a molecule of guanosine triphospahte (GTP) is hydrolyzed so that eIF2 is recycled to its active form of eIF2-GTP [16]. This allows eIF2-GTP to continue with the initial step of protein synthesis. Once eIF2-GTP is released, the second step can occur. A ribosomal binding site/translation start site forms once eukaryotic initiation factor 4F (eIF4F) recognizes the molecule [16]. The eIF4F complex binds the eukaryotic initiation factor 4E (eIF4E) subunit of eIF4F to the m^7^GTP cap structure present in all eukaryotic mRNAs [16]. Replication of the mRNA strand occurs, thus indicating protein synthesis. The processes of protein synthesis appear to be highly regulated by the amino acid leucine [10-14].

Leucine plays a role in muscle protein synthesis mostly through stimulation of the mammalian target of rapamaycin (mTOR) signaling pathway [15,17,18]. Leucine interacts with two mTOR regulatory proteins, mTOR raptor (or raptor) and rashomolog enriched in the brain (or Rheb) [19,20]. The importance of the regulation of mTOR is that when activated, it phosphorylates the proteins eIF4E binding protein 1 (4E-BP1) and ribosomal protein S6 kinase (S6K1) complex [21,22]. When 4E-BP1 is phosphorylated, it becomes inactive, which allows the continuation of the second step initiation phase of translation by inhibiting its binding to eIF4F complex [10]. This allows additional translation to occur. When S6K1 is phosphorylated, it produces additional eIFs which increases the translation of mRNAs that encode components of the protein synthesis pathway [10,12].

Leucine has been indicated as the sole stimulator of protein synthesis [10-15]. For example, Dreyer et al. conducted a study on 16 young, healthy untrained men to determine the effects of post-workout consumption of either no beverage or leucine-enhanced EAAs [15]. Those consuming the leucine-enhanced beverage one hour following a single bout of resistance exercise had greater rates of protein synthesis than did the control group. Another study conducted by Koopman et al. [23] concurs with the findings of Dreyer. Eight untrained men were randomly assigned to consume one of the three beverages: carbohydrates, carbohydrate and protein or carbohydrate, protein and free leucine following 45 minutes of resistance exercise. The results indicated that whole body net protein balance was significantly greater in the carbohydrate, protein and leucine group compared with values observed in the carbohydrate and protein and carbohydrate only groups, indicating the ability of leucine to augment protein synthesis [23].

Leucine alone appears to be nearly as effective in stimulating protein synthesis as when all branched chain amino acids (BCAAs) are consumed [24-26]. Leucine also seems to have both insulin-dependent and insulin-independent mechanisms for promoting protein synthesis [27,28]. Approximately 3 to 4 g of leucine per serving is needed to promote maximal protein synthesis [29,30]. See Table 2 for the leucine content of protein sources for all protein ingestion timing studies referenced in this review.

**Table 2:** Leucine content of protein sources for studies that used a protein ingestion timing method

**Research study**	**Protein used**	**Leucine content**	**Reached 3g Threshold for Leucine**
Hoffman et al. [31]	42 g of a proprietary blend of protein (enzymatically hydrolyzed collagen protein isolate, whey protein isolate, and casein protein isolate)	3.6 g	Yes
Hoffman et al. [32]	42 g of a proprietary protein blend (enzymatically hydrolyzed collagen protein isolate, whey protein isolate, casein protein isolate, plus 250 mg of additional branch chain amino acids)	3.6 g	Yes
Cribb et al. [33]	Whey protein, creatine and dextrose mixture based on individuals bodyweight	3.49 g^1^	Yes
Verdijk et al. [34]	20 g of casein split into two 10 g servings pre- and post-workout	1.64 g total in 2 servings^2^	No
Hulmi et al. [35]	30 g whey split into two 15 g servings pre- and post-workout	3.4 g total in 2 servings	No as only 1.7 g were given at a time
Andersen et al. [36]	25 g of a protein blend (16.6 g of whey protein; 2.8 g of casein; 2.8 g of egg white protein; and 2.8 g of l-glutamine)	2.29 g ^2,3^	No
Elliot et al. [37]	237 g of whole milk	0.639 g	No
Hartman et al. [38]	500 mL of fat-free milk	1.35 g	No
Wilkinson et al. [39]	500 mL of fat-free milk	1.35 g	No
Rankin et al. [40]	Chocolate milk based on bodyweight	Unknown	Unknown
Josse et al. [41]	500 mL of fat-free milk	1.35 g	No

^1^ 3.49 g is based on the amount of leucine that the mean weight (80 kg) of the participants in this study.

^2^ Leucine content of casein received from Tang et al. [42].

^3^ Leucine content of egg white received from Norton et al. [43].

### Types of protein

There are numerous protein sources available to the consumer. This review article focuses on studies that have used a variety of dairy- and soy-based protein sources. This section describes each of these protein sources and compares their quality on the two scales most relevant to this review: biological value and protein digestibility corrected amino acid score (PDCAAS) [44]. Biological value (BV), determines how efficiently exogenous protein leads to protein synthesis in body tissues once absorbed, and has a maximum score of 100 [44]. PDCAAS numerically ranks protein sources based on the completeness of their essential amino acid content, and has a maximum score of 1.0 [44]. The BV and PDCAAS are both important in understanding bioavailability and quality of different protein sources.

Three sources of dairy protein typically used in studies of muscle hypertrophy and strength are bovine milk, casein and whey. Bovine milk is a highly bioavailable source of protein, comprising 80% casein and 20% whey [44]. Overall, bovine milk has a BV of 91 and a PDCAAS of 1.00 indicating that it is readily absorbed by the body, promoting protein synthesis and tissue repair, and provides all essential amino acids (EAAs). Casein, with a BV of 77 and a PDCAAS of 1.00, is the predominate protein in bovine milk and gives milk its white color [44]. It exists in micelle form, and within the stomach will gel or clot, thus resulting in a sustained release of amino acids [45]. Compared with milk, it is less bioavailable, but like milk, it provides all EAAs. Whey the other protein found in milk, is the liquid part of milk that remains after the process of cheese manufacturing [44]. With a BV of 104 and a PDCAAS of 1.00, whey is superior to both milk and casein. It contains all EAAs, and its excellent bioavailability leads to rapid protein synthesis [44,45].

Soy is a vegetable-based protein source that is useful for vegetarians and individuals who are lactose- or casein-intolerant. Soy has a BV of 74 and PDCAAS of 1.00, indicating that it is not as bioavailable as milk based protein, but does contain all EAAs [44].

### Whole-food protein intake studies: post workout only

The timing of protein intake has been an important condition in studies on muscle hypertrophy and strength in weight-trained individuals. In this section, studies using whole-food protein sources (i.e. bovine and soy milk) have been reviewed with respect to their intake following weight-resistance training.

Many studies on the effects of protein intake timing on physical changes have used protein supplements [31-36], but some studies have used milk and other fluid protein sources. In a study focused on protein intake following a single resistance training session, Elliot et al. examined milk consumption post-workout in 24 untrained men and women [37]. Subjects were randomly assigned to one of three groups: 237 g of fat-free milk, 237 g of whole milk, or 393 g of isocaloric fat-free milk. The findings indicated that in untrained individuals, threonine uptake was significantly higher for those consuming 237 g whole milk versus those consuming 237 g fat free milk. Threonine uptake is indicative of net muscle protein synthesis. The results of this study suggest that whole milk increased utilization of available amino acids for protein synthesis [37]. Tipton et al. conducted a study on 23 untrained men and women in which participants ingested 1) 20 g casein, 2) 20 g whey, or 3) artificially sweetened water one hour following heavy leg resistance exercise [46] Positive changes in net muscle protein balance resulted for both protein groups but not for the control group. This study indicated that milk proteins (both casein and whey) post-workout increased protein synthesis [46].

Various studies have compared whole-food protein sources to determine which is most effective in improving muscle mass and strength gains. Hartman et al. conducted a study comparing the use of milk, soy protein, or carbohydrate drinks by 56 young untrained males [38]. Subjects were assigned to one of three groups; each consumed 500-milliliter (mL) of a) fat-free milk, b) an isocaloric, isonitrogenous, and macronutrient- matched soy-protein beverage, or c) an isocaloric carbohydrate beverage immediately following and again one hour after resistance exercise. Body composition, muscle hypertrophy, and strength measurements were recorded at baseline and three days following 12 weeks of training 5 d.wk^-1^. The group using milk post-workout had significantly increased body weight and decreased body fat versus the other two groups, indicating an increase in lean body mass (LBM). Results indicated that consumption of fat-free milk post-workout was statistically more effective than soy protein in promoting increases in LBM (p<0.01), increases in type II muscle fiber area (p<0.05) and decreases in body fat (p<0.05) [38]. These results were similar to those found by Wilkinson et al. [39]. Researchers assigned eight weight-trained men to either 500 mL of skim milk or an isonitrogenous, isocaloric, and macronutrient-matched soy-protein beverage following resistance exercise [39]. A crossover design was used so that all participants consumed either milk or soy on their first trials and alternated to the other supplement on the second trials. Trials were separated by one week. Both protein drinks increased protein synthesis and promoted increases in muscle mass; however, the consumption of skim milk had a significantly greater impact on the development of muscle mass than did consumption of the soy protein [39]. Both Hartman et al. [38] and Wilkinson et al. [39] demonstrated the superiority of milk proteins over soy protein in building muscle mass. This may be due to the fact that soy has a lower BV than milk (74 versus 91 respectively), resulting in lower bioavailability, thus providing less protein synthesis in body tissues.

Rankin et al. studied the effects of milk versus carbohydrate consumption post-resistance exercise on body composition and strength [40]. Nineteen untrained men were randomly assigned to one of two groups that provided 5 kcal^.^kg^-1^ body weight of either chocolate milk, or a carbohydrate-electrolyte beverage. Subjects completed whole body dual-energy X-ray absorptiometry (DXA) scans and strength assessments prior to and after following a 3 d^.^wk^-1^ for 10-weeks weightlifting protocol. Results indicated that both groups had increases in LBM and strength, but there were no significant between-group differences [40]. The addition of a control group to this study would have helped determine whether increases in strength were due solely to the weightlifting program or to the combination of exercise and supplementation. The findings suggest that consumption of chocolate milk post-exercise may be effective in increasing LBM in weightlifters, but more studies using control groups are needed.

Milk consumption and resistance training also have been investigated in women. Josse et al. examined the effects of milk consumption post-workout on strength and body composition in 20 healthy untrained women [41]. Subjects were assigned to 500 mL of either fat-free milk or isocaloric maltodextrin. The women followed a weight training protocol 5 d^.^wk^-1^ for 12-weeks. Each participant completed strength assessments, DXA scans, and blood tests. The group consuming milk had statistically greater increases in LBM, greater fat mass losses and greater gains in strength, providing evidence that fat-free milk consumption post-workout was effective in promoting increased LBM and strength in women weightlifters [41]. The results of this study support those of previous studies completed in men showing that milk consumption post-workout has a favorable effect on MPS [37-40].

### Protein supplement intake studies: a comparison of timing protocols

Protein and amino acid supplements have been used widely in studies showing their effectiveness on protein synthesis. Hoffman et al. compared protocols providing protein supplementation and subsequent effects on muscle strength and body composition in 33 strength-trained adult men [31]. Two protein-intake timing strategies were implemented over the course of 10-weeks of resistance weight-training [31]. One group consumed a protein supplement comprising enzymatically hydrolyzed collagen-, whey-, and casein-protein isolates pre/post-workout. A second group consumed the same supplement in the morning upon awakening and in the evening. A control group was not given the protein blend. The average caloric intake of the three groups was 29.1 ± 9.7 kcal^.^kg body mass-^1.^d^-1^.

Muscle strength was assessed through one-repetition maximum (1RM) on bench and leg press. Body composition was assessed using DXA [31]. There were no group differences in body composition based on timing of supplementation [31]. All groups increased the 1RM for squats, indicating increased muscle strength. Only the protein supplement groups also showed significant increases in the 1RM for bench press, indicating improved strength [31]. These findings indicated that supplementation was beneficial for increasing muscle strength in 1 RM bench press but timing of ingestion was not important. The results on body composition may have had different effects if participants had consumed adequate kcal^.^kg^-1^, as greater-than-maintenance-caloric needs are required for muscular hypertrophy to occur. Strength did increase, providing evidence to both the effectiveness of protein supplementation on strength and the effectiveness of the workout regimen used in this study. Future studies should ensure that participants are consuming greater than 44–50 kcal^.^kg^-1^ to maximize muscle hypertrophy [9].

Hoffman et al. conducted a double-blind study focusing on the use of protein supplements to hasten recovery from acute resistance weight training sessions [32]. Fifteen strength-trained men were matched for strength then randomly assigned to receive 42 g of either a) a proprietary protein blend (enzymatically hydrolyzed collagen-, whey-, or casein-protein isolates, plus 250 mg of additional BCAA pre and post workout), or b) a placebo of maltodextrin pre-and post-workout [32]. Participants initially performed a 1RM for squat, dead lift, and barbell lunge exercises. On the second visit, subjects performed four sets of at least 10 repetitions at 80% of their 1RM for the exercises with 90 seconds between sets. On visits three (24 hours from visit two) and four (48 hours from visit two), participants performed four sets of squats with the previous weight and performed as many repetitions per set as possible [32]. Hoffman et al. [32] found that the group receiving the proprietary protein blend performed significantly more repetitions at visits three and four than did subjects receiving the placebo. These findings provide evidence that protein supplementation pre- and post-workout is useful in maximizing weight-training performance, as well as in hastening exercise recovery 24 and 48 hours post-exercise.

Timing of supplementation in relation to the resistance workout also has been studied [33]. Cribb et al. assigned 23 male bodybuilders to one of two groups: those who received a supplement a) before and after a workout, or b) in the morning and evening. The supplement contained 40 g protein (from whey isolate), 43 g carbohydrate (glucose), and seven g creatine monohydrate per 100 g. Each participant was given the supplement in quantities of 1.0 g^.^kg^-1^ body weight. All participants followed a preliminary resistance weight-training program for 8–12 weeks before baseline measurements were taken. Participants then started the 10-week resistance weight-training session which was divided into three distinct stages: preparatory (70–75% 1RM), overload phase 1 (80–85%1RM), and overload phase 2 (90–95% 1RM) [33].

Results indicated significant differences in body composition in the group consuming the supplement pre- and post-workout [33]. This group experienced increased LBM and decreased body fat. Both groups demonstrated increases in strength, but the pre- and post-workout group demonstrated significantly greater gains [33], indicating that timing of the ingestion of the protein supplement was crucial. This is contradictory to the findings of Hoffman et al. [31] with respect to changes in body composition. This could be because Cribb et al. [33] used a supplement that was a combination of protein, carbohydrate and creatine whereas, Hoffman et al. [31] supplemented with protein only. The major finding of this study was that after 10 weeks of training, supplementation pre/post each workout resulted in greater improvements in 1RM strength and body composition (increased LBM and decreased body fat percentage) compared with a matched group who consumed supplement in the morning and evening, outside of the pre- and post-workout time frames.

The majority of studies of protein intake and resistance exercise have been conducted on younger adult males [31-33]. In contrast, Verdijk et al. investigated the impact of the protein, casein hydrolysate, on muscle hypertrophy in healthy untrained elderly men [34]. Researchers randomly assigned 28 elderly men to consume either a protein supplement or a placebo pre- and post-workout. Subjects performed a 12-week resistance weight-training program requiring weightlifting 3 d ^.^wk^-1^. Baseline and ending measurements were obtained, including strength assessments, CT scans, DXA scans, blood samples, 24-hour urine samples, muscle biopsies, and immunohistochemistry tests. Results indicated no differences in ending measurements between the protein group and placebo group in muscle hypertrophy, strength, or body composition [34], suggesting that for elderly men, intake of 20 g casein hydrolysate before and after resistance training does not increase muscle hypertrophy or strength. In this study, however, only 20 g of casein was used, and it was divided into two servings. This protocol would not have provided participants with the required 3 g of leucine needed to maximize protein synthesis. Additionally, since casein is slow digesting [44,45], it may not have been ideal for use in a study of elderly men. Future studies with this population should incorporate whey protein, which is highly bioavailable in an amount that would provide at least 3 g leucine [29,30]. Studies comparing the effects of supplementation with adequate protein and those with creatine-enhanced protein pre-and post-workout also should be conducted to determine whether creatine is needed to produce the desired outcomes, as has been demonstrated in younger men [33] (See Table 2).

The long-term use of whey protein pre- and post- resistance exercise was investigated by Hulmi et al. [35], by assigning participants to one of three groups:1) 15 g of whey protein before and after resistance exercise, 2) a placebo before and after resistance exercise, or 3) no supplement no participation in weightlifting but continued habitual exercise as they did prior to the study. Participants in the first two groups completed two resistance exercise sessions per week for 21 weeks consisting of both upper and lower body multi-joint lifts. All participants then had biopsies performed on their vastus lateralis. Results indicated that the whey protein group had significantly greater increases than the other groups in vastus lateralis hypertrophy, and greater overall muscle hypertrophy [35]. These findings provide evidence that whey protein supplementation pre- and post-workout is useful in increasing muscle hypertrophy.

Andersen et al. examined the effects of a mixed blend of proteins on muscle strength and muscle fiber size [36]. They studied the ingestion pre- and post-workout of 25 g of a protein blend (whey, casein, egg-white proteins, and l-glutamine), compared with a maltodextrin supplement, over the course of a 3 d^.^wk^-1^ 14-week resistance-training program. Results of muscle biopsies from the vastus lateralis indicated that the protein supplementation group had greater increases in muscle hypertrophy and in squat jump height [36]. Results of this study provide evidence that supplementation with a blend of whey, casein, egg-white proteins, and l-glutamine pre- and post-workout helps promote muscle hypertrophy and improved physical performance.

### Training effects

The effects of training protocols also are very important on increases in strength and muscle hypertrophy. All studies used in this review followed a resistance weight-lifting protocol [31-36,38-41]. It appears from the studies referenced in this review that a training protocol tailored for muscle hypertrophy and strength should be at least 10–12 weeks in length and involve three to five training sessions weekly, consisting of compound lifts that include both the upper and lower body [31,33,35,36,38,40,41].

## Conclusions

Researchers have tested the effects of types and timing of protein supplement ingestion on various physical changes in weightlifters. In general, protein supplementation pre- and/or post-workout increases physical performance [31-34,38-41], training session recovery [32], lean body mass [33,38-41], muscle hypertrophy [35,38-41], and strength [31,33,38,40,41]. Specific gains, however, differ based on protein type and amounts [31-36]. For example, whey protein studies showed increases in strength [31,33], whereas, supplementation with casein did not promote increases in strength [34]. Additional research is needed on the effects of a protein and creatine supplement consumed together, as one study has shown increases in strength and LBM [33].

Studies on timing of milk consumption have indicated that fat-free milk post-workout was effective in promoting increases in lean body mass, strength, muscle hypertrophy and decreases in body fat [38-41] Milk proteins have been shown to be superior to soy proteins in promoting lean body mass [38] and muscle mass development [39]. What is interesting about the milk studies [38-41] is that not one of them provided the 3–4 g of leucine needed to promote maximal MPS (See Table 2), yet they all showed improvements in LBM and strength. This raises the question of whether other components in milk could have contributed to the changes observed. Future researchers should investigate whether other properties of milk help increase LBM when leucine intake is suboptimal to provide maximal MPS. Researchers should also investigate the effects of protein supplements when participants are consuming adequate kcal^.^kg^-1^ and g^.^kg^-1^ of protein to maximize muscle hypertrophy.

The effects of timing of ingestion of EAAs on physical changes following exercise also have been studied [47,48]. Tipton et al. [47] found that the ingestion of EAAs prior to resistance exercise was more beneficial than post-ingestion in promoting protein synthesis [47], but these results did not hold true with respect to whey protein ingestion [48]. Once a protein has been consumed by an individual, anabolism is increased for about three hours postprandial with a peak at about 45–90 minutes [14]. After about three hours postprandial, MPS drops back to baseline even though serum amino acid levels remain elevated [14]. These data show that there is a limited time window within which to induce protein synthesis before a refractory period begins. With this in mind, an ideal protein supplement after resistance exercise should contain whey protein, as this will rapidly digest and initiate MPS, and provide 3–4 g of leucine per serving, which is instrumental in promoting maximal MPS [29,30]. A combination of a fast-acting carbohydrate source such as maltodextrin or glucose should be consumed with the protein source, as leucine cannot modulate protein synthesis as effectively without the presence of insulin [27,28] and studies using protein sources with a carbohydrate source tended to increase LBM more than did a protein source alone [33,37-41]. Such a supplement would be ideal for increasing muscle protein synthesis, resulting in increased muscle hypertrophy and strength. In contrast, the consumption of essential amino acids and dextrose appears to be most effective at evoking protein synthesis prior to rather than following resistance exercise [47]. To further enhance muscle hypertrophy and strength, a resistance weight-training program of at least 10–12 weeks 3–5 d ^.^wk^-1^ with compound movements for both upper and lower body exercises should be followed [31,33,35,36,38,40,41].

## Abbreviations

ml: Milliliter; d: Day; g: Gram; kg: Kilogram; wk: Week; RDA: Recommended dietary allowance; LBM: Lean body mass; MPS: Maximal protein synthesis; 1RM: One-repetition maximum; DXA: Dual-energy X-ray absorptiometry; CT: Computed tomography; kcal.kg^-1^: Kilocalories per kilogram; g.kg^-1^: Gram per kilogram; BCAA: Branched chain amino acids; EAA: Essential amino acids; Hr: Hour; methionyl-tRNA: Methionyl-transfer ribonucleic acid; 40S: Eukaryotic small ribosomal subunit; 43S: Pre-initiation complex; eIF2: Eukaryotic initiation factor 2; mRNA: Messenger ribonucleic acid; GTP: Guanosine triphosphate; eIF4F: Eukaryotic initiation factor 4F; eIF4E: Eukaryotic initiation factor 4E; mTOR: Mammalian target of rapamycin; raptor: mTOR raptor; Rheb: Rashomolog enriched in the brain; 4E-BP1: eIF4E binding protein 1; S6K1: Ribosomal protein S6 kinase.

## Competing interests

The authors declare that they have no competing interests.

## Authors' contributions

MS was the primary author of the manuscript. JL, AP and AS played an important role in manuscript preparation and revisions. All authors have read and approved the final manuscript.
